# Apolipoprotein E knockout may affect cognitive function in D-galactose-induced aging mice through the gut microbiota–brain axis

**DOI:** 10.3389/fnins.2022.939915

**Published:** 2022-09-15

**Authors:** Bowei Chen, Jian Yi, Yaqian Xu, Huiqiao Wen, Fengming Tian, Yingfei Liu, Lan Xiao, Lisong Li, Baiyan Liu

**Affiliations:** ^1^The First Affiliated Hospital, Hunan University of Chinese Medicine, Changsha, China; ^2^College of Pharmacy, Hunan University of Chinese Medicine, Changsha, China; ^3^College of Information Science and Engineering, Hunan University of Chinese Medicine, Changsha, China; ^4^Hunan Academy of Chinese Medicine, Changsha, China

**Keywords:** apolipoprotein E, aging, cognitive function, gut microbiota, metabolomics, oxidative stress

## Abstract

The gut microbiota plays an important role in central nervous system (CNS) disorders. Apolipoprotein E (ApoE) can affect the composition of the gut microbiota and is closely related to the CNS. However, the mechanism by which ApoE affects cognitive dysfunction through the gut microbiota–brain axis has thus far not been investigated. In this study, we used wild-type mice and ApoE knockout (ApoE^–/–^) mice to replicate the aging model and examined the effects of ApoE deletion on cognitive function, hippocampal ultrastructure, synaptophysin (SYP) and postsynaptic density 95 (PSD-95) in aging mice. We also explored whether ApoE deletion affects the gut microbiota and the metabolite profile of the hippocampus in aging mice and finally examined the effect of ApoE deletion on lipids and oxidative stress in aging mice. The results showed that the deletion of ApoE aggravated cognitive dysfunction, hippocampal synaptic ultrastructural damage and dysregulation of SYP and PSD-95 expression in aging mice. Furthermore, ApoE deletion reduced gut microbial makeup in aging mice. Further studies showed that ApoE deletion altered the hippocampal metabolic profile and aggravated dyslipidemia and oxidative stress in aging mice. In brief, our findings suggest that loss of ApoE alters the composition of the gut microbiota, which in turn may affect cognitive function in aging mice through the gut microbiota–brain axis.

## Introduction

Aging is a process in which the functions of various tissues and organs in the body gradually degenerate. With the intensification of population aging, aging has become a serious social problem (Joe and Ringman, 2019). Brain aging-induced cognitive and memory decline is one of the early symptoms of aging patients, and changes in hippocampal structure and function are closely related to learning and memory impairments (Kodali et al., 2021; Kokudai et al., 2021). At present, effectively delaying brain aging, maintaining the normal function of the hippocampus, and preventing cognitive dysfunction have become research hotspots in medicine.

Recently, increasing clinical and experimental evidence has suggested that the gut microbiota plays a crucial role in central nervous system (CNS) diseases through the gut microbiota-brain axis (Chakrabarti et al., 2022; Mou et al., 2022). The cognitive dysfunction associated with aging has been reported to be associated with intestinal microbiome disturbances (Pw and Jeffery, 2015; Mangiola et al., 2018). For example, medical studies in the elderly population have found higher *Firmicutes*/*Bacteroidetes* ratios in the gut microbiome of demented subjects than in non-demented controls (Saji et al., 2019). The gut microbiota is considered an invisible organ that mediates bidirectional signaling between the gut-brain axis (Doifode et al., 2021). Unfortunately, the relevant mechanisms between gut microbiota and brain aging have not been fully elucidated. Therefore, in-depth studies are warranted to elucidate the potential link between the gut microbiota-brain axis and cognitive impairment induced by brain aging.

Apolipoprotein E (ApoE) is the main plasma apolipo- protein, and it regulates lipid metabolism and maintains cholesterol balance. It also participates in the normal growth and development and damage repair of the CNS (Aires et al., 2021). Studies have shown that ApoE is the most abundantly expressed apolipoprotein in the brain and is responsible for regulating a large part of brain lipid metabolism, especially the transfer of cholesterol and phospholipids from glial cells to neurons (Hudry et al., 2019). Furthermore, loss of ApoE disrupts the blood–brain barrier in aging mice (Mulder et al., 2001) and leads to cognitive impairment (Zerbi et al., 2014) and cerebrovascular dysfunction (Bell et al., 2012). Recent studies have also found that ApoE deficiency alters the composition of the gut microbiome (Gan et al., 2022; Zajac et al., 2022). However, the mechanism by which ApoE affects lipid metabolism and cognitive impairment through the gut microbiota brain axis has not been investigated to date.

The gut microbiota is both a participant and a regulator of metabolic processes (Wang et al., 2017). Metabolomics can be used to effectively screen biomarkers and deeply analyze the molecular mechanisms of host health or disease (Rowland et al., 2018). To this end, we used ApoE knockout (ApoE^–/–^) mice as study subjects to explore whether ApoE is involved in postaging cognitive dysfunction through the gut microbiota-brain axis. We explored the effect of ApoE deletion on cognitive function and gut microbes in D-galactose-induced aging mice. We also used a metabolomic approach to confirm whether the absence of ApoE affects the metabolite profile of the aging mouse hippocampus. In addition, we examined the effect of ApoE deletion on blood lipids and oxidative stress in aging mice. We hope to reveal the mechanism by which ApoE affects cognitive dysfunction in aged mice through the gut microbiota-brain axis.

## Materials and methods

### Animals

Twenty 10-week-old male SPF ApoE^–/–^ mice were purchased from Gempharmatech Co., Ltd. (Nanjing, China), with a body weight of 25 ± 5 g [serial number: T001458, genetic background: C57BL/6 J, genotype: (ApoE) KO/KO, and license number: CXK (SU) 2018-0008]. Forty 10-week-old male SPF wild-type C57BL/6J mice, with a body weight 20 ± 2 g, were also used. Bedding materials and feed were provided by Hunan Laike Jingda Co., Ltd. (Changsha, China). The animals had free distilled drinking water, and the housing conditions were as follows: temperature of 20–25°C, humidity of 40–55%, natural light, normal feeding, and bedding replacement every other day.

### Main reagents and instruments

D-galactose (V900922) was purchased from Sigma–Aldrich (Shanghai, China), anti-synaptophysin (SYP) antibody (ab32127) and anti-postsynaptic density 95 (PSD-95) antibody (ab238135) were purchased from Abcam (Cambridge, United Kingdom), and a superoxide dismutase (SOD) detection kit (20190412), a glutathione peroxidase (GSH-PX) detection kit (20190309), and a malondialdehyde (MDA) detection kit (20190315) were purchased from Jiancheng Co., Ltd. (Nanjing, China).

Vectra3 tissue section analysis system (Marlborough, United States), Illumina NovaSeq 6,000 sequencing system (Santiago, United States), Thermo Dionex U3000 UHPLC (Waltham, United States), Agilent 7890B-5977B GC–MS (Santa Clara, United States), Enspire multifunctional microplate reader (Waltham, United States), Waters ACQUITY UPLC HSS T3 (100 mm × 2.1 mm, 1.8 μm) chromatographic column (Framingham, United States).

### Design of animal experiments

**FIGURE 1:**
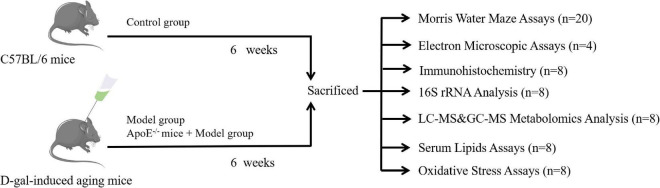
Experimental design.

Forty wild-type mice were randomly divided into two groups [the control group and the model group (*n* = 20)], after adaptive feeding, and the other 20 ApoE^–/–^ mice were set as the ApoE group. Except for the control group, the other groups were injected subcutaneously with 100 mg⋅kg^–1^ of D-galactose on the back of the neck once a day for 6 consecutive weeks, and the blank group was injected with the same amount of normal saline. The subcutaneous injection of D-galactose is a commonly used method to replicate aging models (Azman and Zakaria, 2019; Huang et al., 2020). After the Y maze and Morris water maze (MWM) in the 6th week, all mice were anesthetized by intraperitoneal injection of 1% sodium pentobarbital, the eyeballs were removed, blood was collected, and then the serum was obtained by centrifugation. Eight mice were randomly selected from each group for analysis of the gut microbiome, metabolomics, blood lipids and oxidative stress, and the remaining 12 were subjected to transmission electron microscopy analysis and immunohistochemical detection. As shown in Figure 1. This experimental protocol was approved by the Ethics Committee of the First Affiliated Hospital of Hunan University of Traditional Chinese Medicine (ZYFY20210710).

### Y–maze test

According to the details in Reference (Suryavanshi et al., 2014), the memory ability of brief learning in mice was evaluated using the Y-maze test. The Y-maze consists of 3 arms radially oriented outward in 3 equal parts, and a single arm is 30 cm in length, 8 cm in width, and 15 cm in height with an angle between arms of 120. The Y maze was placed in a quiet room, and at the midpoint of the Y-maze at the beginning of the experiment, the movements of the mice were recorded over 8 min, and only consecutive entries into 3 different arms were counted as 1 correct alternating exploration, after which the mice were returned to their cages. After each assay was finished, a 75% ethanol wipe was used to eliminate mouse odor. The alternation rate was calculated as follows: alternation rate = number of correct alternations/(total alternations-2) × 100%.

### Morris water maze test

According to the details in Hui et al. (2017), the MWM was used to test the long-term working memory ability of the mice. The MWM used a black round stainless steel pool (depth 60 cm, diameter 150 cm), and the top of the pool was connected to a video detection system. The platform was fixed in the second quadrant, tap water was injected into the pool, the water level was 1–2 cm higher than the platform, and the water temperature was controlled at 22–24°C. The mice were put into the water with their heads facing the pool wall, the water entry points were randomly selected, and the detection time was 60 s. The time when the mouse found the underwater platform was recorded. If it was found within 60 s, the animal was allowed to stay on the platform for 10 s to rest. If the platform was not found, the animal was guided to the platform and stayed for 10 s. Each animal was trained three times a day, with an interval of 15–20 min between each training session for 5 consecutive days. On the 6th day, the platform was removed, the animals were placed into the water from the opposite side of the original platform quadrant, and the latency and number of times the animals crossed the original platform quadrant within 60 s were recorded.

### Detection of synaptic ultrastructure in the hippocampus

Specimens that had been placed in the transmission electron microscope (TEM) for 48 h were postfixed in 1% osmic acid at room temperature for 2 h, dehydrated stepwise through an ethanol gradient, and again dehydrated in acetone. After embedding in an embedding machine, the tissues were cut into 60–80-nm ultrathin sections, double stained with uranyl acetate and lead citrate, and dried overnight at room temperature. Synapses with clearly observed presynaptic and postsynaptic membranes and synaptic vesicles were selected and photographed through TEM. Five synapses were randomly selected from each mouse, and a total of 20 synapses were selected from each mouse group, and structural parameters of synaptic interfaces were calculated using Image-Pro Plus 6.0 software (Jones and Devon, 1978; Güldner and Ingham, 1980).

### Immunohistochemical detection

The expression levels of SYP and PSD-95 were determined by immunohistochemistry. The detection procedure was similar to that described in a previous study (Chen et al., 2022): the sections were dewaxed, antigen retrieval and blocked sequentially. SYP (1:100) and PSD-95 (1:100) primary antibodies were added and incubated overnight at 4°C, and then incubated with the corresponding secondary antibodies for 1 h at room temperature. Routine DAB staining was followed by counterstaining with hematoxylin and mounting of the slides. A smart tissue section imaging system was used for whole slide scanning, and images of the dentate gyrus (DG), CA1, and CA3 regions were collected from the hippocampus region. Densitometry was calculated using Image-Pro Plus 6.0 software.

### Gut microbiota analysis

The genomic DNA of mouse cecal contents was extracted using a DNA extraction kit, and then the concentration of DNA was detected by agarose gel electrophoresis and a NanoDrop2000. Using genomic DNA as a template, primers 343F and 798R were used to amplify the V3-V4 region by PCR. After purification and quantification, a sequencing library was constructed. The following sequences were used: V3–V4 forward primer, 343F TACGGRAGGCAGCAGCAG; reverse primer, 798R AGGGTATCTAATCCT.

Using the QIIME 2 analysis process, DADA2 was used to denoise the raw data, cluster with 100% similarity, remove and correct low-quality sequences, identify and dechimerize algorithms, etc. The representative sequences of amplicon sequence variants (ASVs) were aligned with the template sequences in the SILVA-132-99 database to obtain the flora information of all ASVs at the levels of microbial phylum, class, order, family, genus, and species classification.

### Metabolomics analysis

#### LC–MS analysis

The hippocampal tissue was removed from the −80°C refrigerator and thawed at 4°C. Then, 30 mg of tissue sample was accurately weighed, 20 μL of internal standard and 600 μL of methanol-water (V:V = 4:1) were added, and the samples were placed in a grinder and ground. Ultrasonic extraction was performed in an ice-water bath for 10 min. After standing at −20°C for 2 h and centrifuging for 10 min (13,000 rpm, 4°C), 150 μL of the supernatant was aspirated with a syringe, filtered through a 0.22-μm filter, and transferred to a sample vial until LC–MS analysis.

The chromatographic column was an ACQUITY UPLC HSS T3 (100 mm × 2.1 mm, 1.8 μm), and the mobile phases were A-water (containing 0.1% formic acid) and B-acetonitrile (containing 0.1% formic acid). The gradient elution program was 0–4 min, 5% B; 4–9 min, 30% B; 8–10 min, 50% B; 10–14 min, 80% B; 14–15 min, 100% B, and 15–16 min, 5% B. The following chromatographic conditions were used: flow rate, 0.35 mL⋅min^–1^; injection volume, 2 μL; column temperature, 45°C. The following were the mass spectrometry conditions: the ion source was electrospray ionization (ESI); the positive and negative ion scanning modes were used for measurement, with spray voltages of 3,800 and −3,000 V, respectively; the capillary temperature was 320°C; the aux gas heater temperature was 350°C; the sheath gas flow rate was 35 Arb; the aux gas flow rate was 8 Arb; the full MS resolution was 70,000; and the mass range was 100–1 200 m/z. To ensure the stability of the entire analysis system, quality control (QC) samples were used for method verification in this experiment. The QC samples were obtained by mixing 10 μL of each normal sample. One QC sample was injected between every 10 samples to assess the stability of the mass spectrometry system.

#### GC-MS analysis

Similar to the above method, 30 mg of hippocampal tissue was accurately weighed, and 20 μL of internal standard and 600 μL of methanol-water (V:V = 4:1) were sequentially added. Then, the sample was placed in a grinder and ground. Next, 120 μL of chloroform was added, and the sample was vortexed for 2 min and then sonicated in an ice-water bath for 10 min. After standing at −20°C for 30 min, the samples were centrifuged for 10 min (13,000 rpm, 4°C), and 150 μL of the supernatant was removed and placed into a glass derivatization bottle. After evaporating the sample with a centrifugal concentrator desiccator, 80 μL of methoxyamine hydrochloride in pyridine (15 mg/mL) was added to a glass derivatized vial. After vortexing for 2 min, the oximation reaction was performed in a shaking incubator at 37°C for 90 min. After removing the sample, 50 μL of bis (trimethylsilyl) trifluoroacetamide (BSTFA) (containing 1% chlorotrimethylsilane); containing 1% trimethylchlorosilane (TMCS) derivatization reagent and 20 μL of n-hexane were added to 10 μL of 10 internal standards, vortexed for 2 min, and reacted at 70°C for 60 min. Finally, the sample was placed at room temperature for 30 min for GC–MS metabolomic analysis.

The chromatographic conditions were as follows: Column, DB-5MS capillary column (30 m × 0.25 mm × 0.25 μm, Agilent J&W Scientific, Folsom, CA, United States); inlet temperature, 260°C; carrier gas, helium, at a volume flow of 1 mL⋅min-1; injection volume, 1 μL, splitless injection; and solvent delay, 6.2 min. The program temperature was as follows: the initial temperature of the column oven was 60°C and kept for 0.5 min; the temperature increased to 125°C at 8°C/min; the temperature was heated to 210°C at 8°C/min; and finally, the temperature was heated to 305°C at 20°C/min and kept for 5 min. The mass spectrometry conditions were as follows: ion source temperature, 230°C; quadrupole temperature, 150°C; and electron energy, 70 eV. The scanning mode was full scan mode (SCAN), and the mass scanning range was m/z 50–500. Both the LC–MS and GC–MS analyses were performed by OE Biotech Co., Ltd. (Shanghai, China).

### Blood lipid detection

One hundred microliters of serum and distilled water were diluted and mixed at 1:1 and then sent to the Laboratory Department of the First Affiliated Hospital of Hunan University of Chinese Medicine. A Beckman Coulter AU680 automatic biochemical analyzer was used to detect the total cholesterol (TC), triglyceride (TG) and low-density lipoprotein (LDL) levels.

### Oxidative stress detection

After rinsing the hippocampal tissue with precooled normal saline, sterile ophthalmic scissors were used to cut the tissue into pieces, and normal saline was added according to the ratio of m (tissue): V (normal saline) = 1 g:9 mL using an automatic homogenizer. The homogenate was made into a 10% homogenate by mass fraction and centrifuged at 1,000 g (4°C) for 15 min, and the supernatant was diluted with an appropriate amount of normal saline to a suitable concentration range. The instructions of the kit were strictly followed to detect SOD and GSH-Px activity and the MDA content in serum and brain tissue.

### Statistical analysis

GraphPad Prism 8.0.2 statistical analysis software was used for statistical analysis of the data. Measurement data are expressed as the mean plus or minus standard error ($\bar{x}$ ± s). One-way analysis of variance (ANOVA) was used for data comparison among multiple groups in the experiment, and the LSD test was used for multiple comparisons. *P* < 0.05 was considered statistically significant.

In the 16S rRNA analysis, Usearch software was used to dechimerize and cluster the data to obtain operational taxonomic units (OTUs) for alpha diversity and beta diversity analyses. Linear discriminant analysis (LDA) was used to estimate the communities or species that had significantly different effects on sample partitioning.

In the metabolomic analysis, partial least squares discriminant analysis (PLS-DA) combined with variable importance of projection (VIP) > 1 and *P* < 0.05 was used to screen differential metabolites. Metabolic pathway enrichment analysis was performed using the Kyoto Encyclopedia of Genes and Genomes (KEGG) database.^1^

## Results

### Apolipoprotein E deletion aggravates cognitive dysfunction in aging mice

**FIGURE 2:**
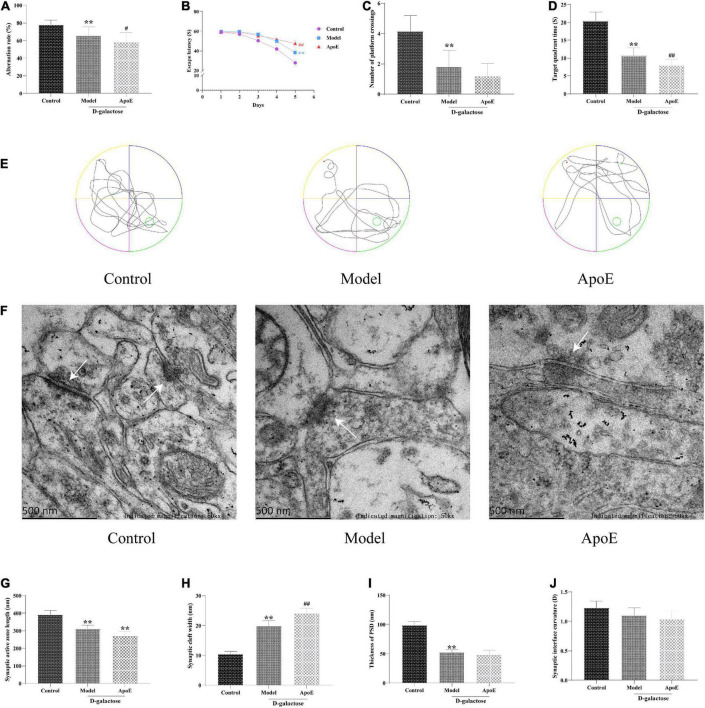
Effects of ApoE on cognitive function and hippocampal synaptic ultrastructure in aging mice. **(A)** Y-maze test, *n* = 20. **(B)** Navigation test, *n* = 20. **(C)** Platform crossing, *n* = 20. **(D)** Target quadrant time, *n* = 20. **(E)** MWM representative figures. **(F)** Ultrastructure of hippocampal synapses. **(G)** Synaptic active zone length, *n* = 4 (Five synapses were randomly selected from each mouse). **(H)** Synaptic cleft width, *n* = 4 (Five synapses were randomly selected from each mouse). **(I)** Thickness of PSD, *n* = 4 (Five synapses were randomly selected from each mouse). **(J)** Synaptic interface curvature, *n* = 4 (Five synapses were randomly selected from each mouse). **p < 0.01 vs. Control group, ^##^*p* < 0.01, ^#^*p* < 0.05 vs. Model group.

In the Y-maze test, compared with the control group, the alternation rate in the model group was significantly decreased (*P* < 0.01), and the alternation rate was further decreased in the ApoE group (*P* < 0.05, Figure 2A). In the navigation test, with an increase in the training time and the number of training sessions, the time for mice in each group to find the platform tended to shorten. Compared with the control group, the escape latency of mice in the model group was significantly prolonged (*P* < 0.01), and the latency of the ApoE group was further prolonged compared with that of the model group (*P* < 0.01, Figure 2B). The results of the space exploration test showed that compared with the control group, the model group spent less time in the target quadrant (*P* < 0.01), and the number of platform crossings was significantly reduced (*P* < 0.01). Compared with that of the model group, there was no significant change in the number of platform crossings in the ApoE group (*P* > 0.05), but the time spent in the target quadrant was significantly increased (*P* < 0.01), as shown in Figures 2C–E. These results suggest that deletion of ApoE aggravates D-galactose injection-induced cognitive impairment.

### Apolipoprotein E deletion aggravates hippocampal synaptic ultrastructural damage in aging mice

TEM was used to assess hippocampal synaptic ultrastructure. The synaptic structure of the hippocampal neurons in the control group was complete, and the presynaptic membrane, synaptic cleft, postsynaptic membrane and postsynaptic dense material were clearly visible. Compared with the control group, the synaptic structure of the model group was blurred, the postsynaptic dense material was sparse, and the boundary between the anterior and posterior membranes was unclear. Compared with that of the model group, the synaptic structure of the ApoE group was blurred, and a large number of vesicles accumulated around these structures, as shown in Figure 2F. In addition, compared with the control group, we observed that the length of the synaptic active zone and the thickness of the postsynaptic density were significantly decreased (*P* < 0.01), and the width of the synaptic cleft was significantly increased (*P* < 0.01) in the model group. Compared with the model group, the length of the synaptic active zone further decreased (*P* < 0.01) and the width of the synaptic cleft further increased (*P* < 0.01) in the ApoE group, but the curvature of the synaptic interface did not alter (Figures 2F–J). This result suggested that the deletion of ApoE aggravated the damage to the hippocampal synaptic structure induced by D-galactose injection.

### Apolipoprotein E deletion aggravates the dysregulation of synaptophysin and PSD-95 expression in the hippocampus of aging mice

**FIGURE 3:**
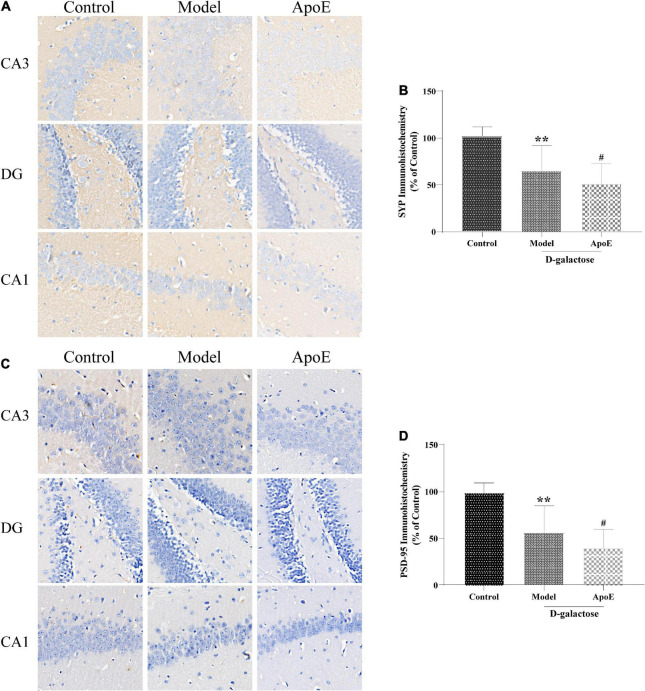
ApoE Deletion aggravates the dysregulation of SYP and PSD-95 expression in the hippocampus of aging mice. **(A)** Immunohistochemistry staining for SYP in different hippocampal regions. **(B)** Quantification of SYP intensity, *n* = 8. **(C)** Immunohistochemistry staining for PSD-95 in different hippocampal regions. **(D)** Quantification of PSD-95 intensity, *n* = 8. **p < 0.01 vs. Control group, ^#^*p* < 0.05 vs. Model group.

Compared with the control group, the expression of SYP and PSD-95 in the hippocampus of the model group was significantly reduced (*P* < 0.01). Compared with the model group, hippocampal SYP and PSD-95 expression were further reduced in the ApoE group (*P* < 0.05). These results suggest that the ApoE deletion aggravates the dysregulation of SYP and PSD-95 expression in the hippocampus induced by D-galactose injection, as shown in Figure 3.

### Deletion of apolipoprotein E affects the gut microbial composition of aging mice

**FIGURE 4:**
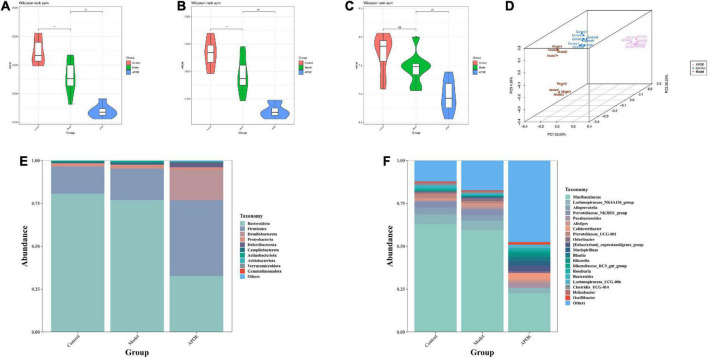
Deletion of ApoE affects the gut microbial composition of aging mice. **(A)** Chao 1 index, *n* = 8. **(B)** Observed species index, *n* = 8. **(C)** Shannon index, *n* = 8. **(D)** PCoA analysis. **(E)** Relative abundance of gut microbiota (phylum level). **(F)** Relative abundance of gut microbiota (genus level). *P < 0.05 vs. Control group, ^##^*p* < 0.01 vs. Model group.

We used the Chao1 index, observed species index and Shannon index to assess the alpha diversity of the gut microbes. We found that the microbiota number and diversity were reduced in the model group compared with the control group (*P* < 0.05). The ApoE group was found to have a further decrease in the number and diversity of microbiota relative to the model group (*P* < 0.01), as shown in Figures 4A–C. In addition, beta diversity analysis was utilized to assess the differences between microbial communities. Principal coordinates analysis (PCoA) based on weighted UniFrac distance showed significantly different gut microbial compositions and structures in each group, as shown in Figure 4D. To understand the impact of ApoE deletion on the gut microbiota, we further analyzed the taxonomic levels of the gut microbiota between the different groups. At the phylum level, compared with the control group, the model group showed that the relative abundance of *Bacteroidota* decreased, the abundance of *Firmicute*s increased, and the ratio of *Bacteroidetes*/*Firmicutes* decreased significantly. Compared with the model group, the ApoE group mice showed decreased relative abundances of *Bacteroidota* and *Desulfobacterota* and increased abundances of *Firmicutes* and *Desulfobacterota*, and the ratio of *Bacteroidetes*/*Firmicutes* decreased significantly, as shown in Figure 4E. At the genus level, compared with the control group, the relative abundance of *Muribaculaceae* decreased and *Parabacteroides* increased in the model group. Compared to the model group, *Muribaculaceae*, *Lachnospiraceae_NK4A136_group* and *Alloprevotella* were significantly decreased in ApoE mice, and the abundances of *Parabacteroides*, *Colidextribacter*, *Mucispirillum*, *Bacteroidesk*, and *Clostridia_UCG-014* were increased, as shown in Figure 4F.

**FIGURE 5:**
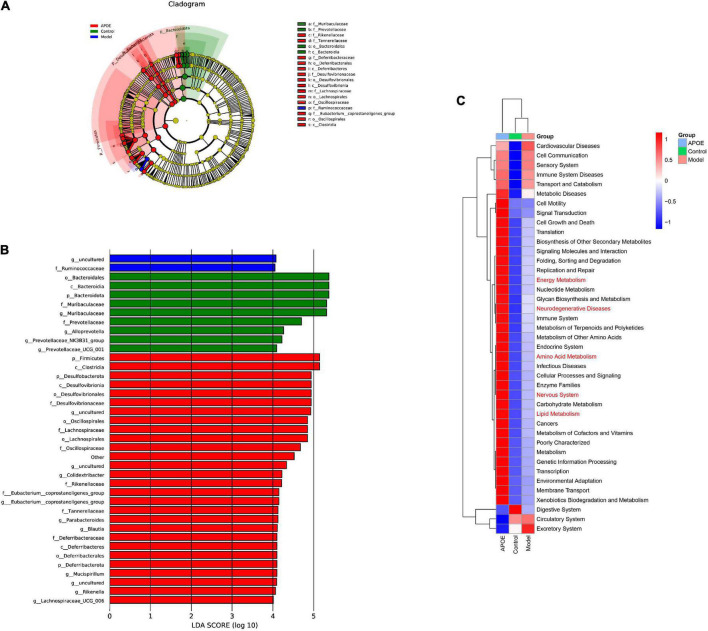
LEfSe and PICRUSt2 analysis. **(A)** Cladogram of LEfSe analysis. **(B)** LDA of LEfSe analysis. **(C)** PICRUSt2 analysis.

Linear discriminant analysis effect size (LEfSe) was applied to identify key microbiota that were differentially represented in ApoE^–/–^ mice. We found that the dominant bacterial groups in the control group were *Bacteroidota* at the phylum level, *Bacteroidia* at the class level, *Bacteroidales* at the order level, *Muribaculaceae* and *Prevotellaceae* at the family level. The dominant bacteria in the model group may be Ruminococcaceae at the family level. The dominant flora of the ApoE group were *Firmicutes* at the phylum level, *Clostridia* at the class level, and *Desulfovibrionaceae Lachnospiracea*e and *Rikenellaceae* at the family level, as shown in Figures 5A,B.

Furthermore, to determine whether taxonomic changes in gut microbes affect their function, functional prediction of representative sequences of gut microbes was performed by PICRUSt2. Compared with the model group, the ApoE group had significant differences in energy metabolism, lipid metabolism, amino acid metabolism, and nervous system pathways, as shown in Figure 5C.

### Deletion of apolipoprotein E alters the metabolic profile of the hippocampus of aging mice

**FIGURE 6:**
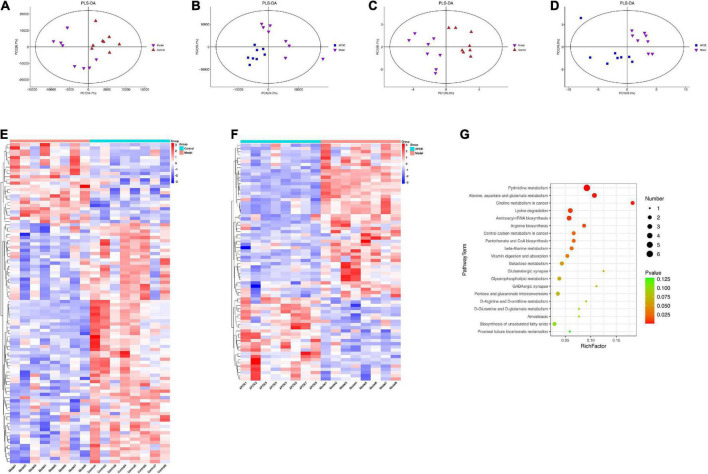
Deletion of ApoE alters the metabolic profile of the hippocampus of aging mice. **(A)** PLS-DA of LC-MS about model group vs. control group. **(B)** PLS-DA of LC-MS about ApoE group vs. model group. **(C)** PLS-DA of GC-MS about model group vs. control group. **(D)** PLS-DA of GC-MS about ApoE group vs. model group. **(E)** Differentially abundant metabolites about model group vs. control group. **(F)** Differentially abundant metabolites about ApoE group vs. model group. **(G)** Analysis of metabolic pathway enrichment.

The metabolite effects of ApoE on the hippocampal tissue of aging mice were first analyzed by LC–MS. We detected a total of 8,251 species peaks, of which 2,872 metabolites were identified. PLS-DA was used to distinguish the overall differences in metabolic profiles between groups. As shown in Figures 6A,B, the differences between the groups were significant. According to the VIP > 1 and *p* < 0.05 criteria, the differential metabolites between the different groups were determined, there were 47 differential metabolites between the model group and the control group, and 32 differential metabolites were found between the ApoE group and the model group (Supplementary Table 1).

Subsequently, we analyzed the metabolite effects of ApoE on the hippocampal tissue of aging mice by GC–MS. GC–MS is efficient at detecting compounds with strong volatility, small molecular weight, and low polarity and can be used as a complement to LC–MS for thermally stable compounds. We identified a total of 668 metabolites by GC–MS. PLS-DA was used to discriminate the overall differences in metabolic profiles between the groups, as shown in Figures 6C,D, and the differences between the groups were significant. Differential metabolites between groups were determined according to VIP > 1 and *p* < 0.05. There were 53 differential metabolites between the model group and the control group, and 42 differential metabolites were found between the ApoE group and the model group (Supplementary Table 2).

By integrating the data of the dual-platform metabolome, we found a total of 100 differential metabolites between the model group and the control group and a total of 74 differential metabolites between the ApoE group and the model group, as shown in Figures 6E,F. Subsequently, we analyzed the metabolic pathways involved in the above 74 metabolites based on the KEGG database. Finally, we found that pyrimidine metabolism, alanine, aspartate, and glutamate metabolism, galactose metabolism and glycerophospholipid metabolism were the main metabolic pathways, as shown in Figure 6G. Notably, we found that alanine, aspartate and glutamate metabolism and glycerophospholipid metabolism, which were significantly enriched by metabolomics, were quite similar to the metabolic pathways such as amino acid metabolism and lipid metabolism predicted by PICRUSt in the 16S analysis. This result suggests that there may be some connection between gut microbes and hippocampal metabolites.

### Deletion of apolipoprotein E alters blood lipids and oxidative stress levels in aging mice

**FIGURE 7:**
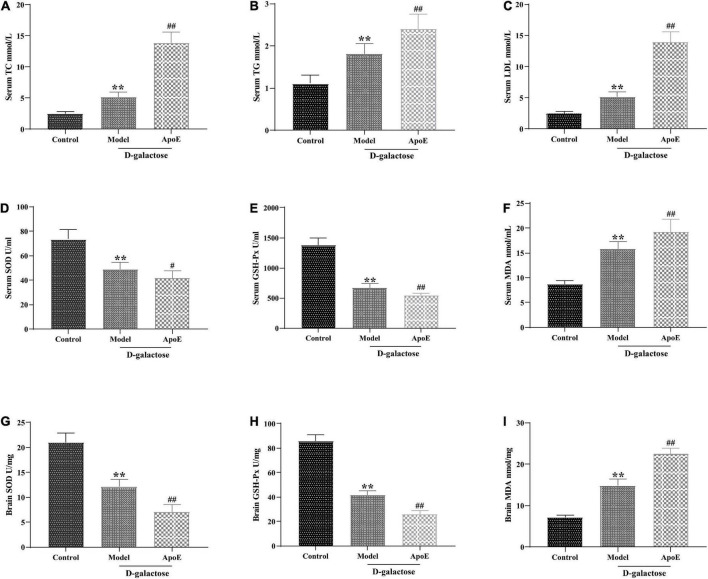
Deletion of ApoE alters blood lipids and oxidative stress levels in rapidly aging mice. **(A)** TC, *n* = 8. **(B)** TG, *n* = 8. **(C)** LDL, *n* = 8. **(D)** SOD in serum, *n* = 8. **(E)** GSH-Px in serum, *n* = 8. **(F)** MDA in serum, *n* = 8. **(G)** SOD in brain tissue, *n* = 8. **(H)** GSH-Px in brain tissue, *n* = 8. **(I)** MDA in brain tissue, *n* = 8. **p < 0.01 vs. Control group. ^#^*p* < 0.05, ^##^*p* < 0.01 vs. Model group.

The enrichment analysis of the above 16 S and differential metabolites in the hippocampus suggested that ApoE deletion might contribute to cognitive impairment in aging mice by affecting lipid metabolism. Therefore, we investigated the effect of ApoE deletion on serum lipids in aging mice. As shown in Figures 7A–C, compared with those of the control group, the concentrations of TC, TG and LDL in the model group were significantly increased (*p* < 0.01). Compared to those of the model group, the levels of TC, TG and LDL in the ApoE group were further increased (*p* < 0.01), suggesting that the absence of ApoE aggravated dyslipidemia in aging mice.

In addition, lipid metabolism disorders often lead to lipid peroxidation, and oxidative stress is an important factor in cellular aging (Mecocci et al., 2018; Zarrouk et al., 2020). We further examined the effect of ApoE deletion on the activities of T-SOD and GSH-Px and the MDA content in the serum and brain tissue of aging mice. As shown in Figures 7D–I, compared with those of the control group, the MDA content in the serum and brain tissue of the mice in the model group was significantly increased (*P* < 0.01), and the activities of T-SOD and GSH-Px were significantly decreased (*P* < 0.01). In comparison with that in the model group, the MDA content in the ApoE group was further increased (*P* < 0.01), and the SOD and GSH-Px activities were further decreased (*P* < 0.05 or *P* < 0.01). This result suggested that ApoE aggravated oxidative stress in aging mice.

## Discussion

At present, studies have confirmed that ApoE is related to atherosclerosis (AS), Alzheimer’s disease (AD) and other vascular diseases and CNS dysfunction. Within the CNS, ApoE is synthesized and secreted by astrocytes and is involved in maintaining the homeostasis of cholesterol and phospholipids, regulating the mobilization and redistribution of cholesterol and phospholipids during neural membrane remodeling, and thus in regulating the maintenance of synaptic plasticity as well as repair processes when neuronal cells are damaged (Cantuti-Castelvetri et al., 2018). Therefore, ApoE^–/–^ mice are not only used in the study of atherosclerosis but are also used as a new animal model in the study of the mechanism of cognitive impairment (Watson et al., 2021). In this study, we found that loss of ApoE aggravated cognitive dysfunction and hippocampal synaptic ultrastructural damage in aging mice, aggravated the dysregulation of hippocampal SYP and PSD-95 protein expression, and altered the gut microbiome composition and the metabolic profile of hippocampal tissue. It was also found that the absence of ApoE aggravated lipid metabolism disorders and oxidative stress in aging mice. Our findings further elucidate the potential of ApoE as a therapeutic target for improving cognitive impairment in aging.

The gut microbiota is considered to be the host’s “second genome” and plays an important role in maintaining body homeostasis and in the development of cognitive dysfunction (Li et al., 2019; Liu et al., 2021b). *Bacteria* have recently been identified in the brains of AD patients, suggesting that the microbiota may be a contributing factor to related neuroinflammation (Emery et al., 2017). Probiotics can modulate gut microbiota dysbiosis and microbiota–gut–brain axis deficits to improve cognitive dysfunction in aged mice (Yang et al., 2020; Liu et al., 2021c). In the present study, Chao1 index and observed species index of α diversity for evaluating intestinal microorganisms in aging mice were significantly decreased, and the correlation index was further decreased after ApoE knockout, suggesting that ApoE knockout reduced gut microbial diversity in aging mice. In addition, at the phylum level, the ratio of *Bacteroidetes*/*Firmicutes* in ApoE^–/–^ mice was significantly decreased. The *Bacteroidetes*/*Firmicutes* ratio is often used to reflect the health of the gut microbiota (Mariat et al., 2009), Recent studies have shown that in amnesic mice, the *Bacteroidetes*/*Firmicutes* ratio was significantly reduced, and restoration of the *Bacteroidetes*/*Firmicutes* ratio reversed memory deficits (Zhao et al., 2022). At the genus level, *Muribaculaceae*, *Lachnospiraceae_NK4A136_group*, *Parabacteroides*, and *Alloprevotella* were significantly decreased in ApoE group mice, and the abundance of *Parabacteroides* was increased. Studies have found that a high abundance of *Muribaculaceae* is closely related to longevity (Sibai et al., 2020); the high abundance of *Lachnospiraceae_NK4A136_group* can improve inflammation and oxidative stress in aging mice (Sheng et al., 2022). *Alloprevotella* is a beneficial bacterium that is closely related to lipid metabolism in aging mice (Wu et al., 2022), and increasing the abundance of *Alloprevotella* can improve the memory function of mice (Liu et al., 2021a). In contrast, *Parabacteroide*s can be used as an independent risk factor for mild cognitive impairment in the elderly (Khine et al., 2020), and a high abundance of *Parabacteroides* can exacerbate neurodegeneration (Blacher et al., 2019). LEfSe analysis found that at the phylum level, the dominant flora was likely *Firmicutes* in ApoE^–/–^ mice. *Firmicute*s are mostly gram-positive bacteria and are significantly increased in the gut of aging mice (Kim et al., 2016). Finally, through PICRUSt2 analysis, we found that energy metabolism, lipid metabolism, amino acid metabolism, and nervous system may be the main metabolic pathways of the differential flora. Based on the above studies, we speculated that ApoE might regulate the ratio of *Bacteroidetes*/*Firmicutes* to affect lipid metabolism and oxidative stress to improve age-related cognitive dysfunction, but the mechanism remains to be further investigated.

A link between metabolic biomarkers and the gut microbiota has been demonstrated, with important consequences for the host (Luo et al., 2020). In this study, the endogenous metabolites in the hippocampus of ApoE^–/–^ aging mice were systematically analyzed based on LC–MS and GC–MS metabolomics platforms. The ApoE group was significantly separated from the model group, suggesting significant changes in hippocampal metabolites. Seventy-four differentially expressed metabolites were identified as potential metabolic markers affected by ApoE. KEGG enrichment analysis found that pyrimidine metabolism; alanine, aspartate and glutamate metabolism; galactose metabolism; and glycerophospholipid metabolism were the main metabolic pathways. At present, the role of pyrimidine metabolism in the aging process remains to be further defined. In addition, some scholars have studied the metabolic changes in the levels of glutamate groups (glutamic acid, gamma-aminobutyric acid, glutamine, aspartic acid and alanine) in the rat brain, and the results show that the changes in the levels of these amino acids are related to aging (Rajeswari and Radha, 1984). Glycerophospholipids are amphiphilic molecules that play important roles in functions such as nerve cell membranes, receptors, transporters, ion channels, and reservoirs for lipid mediators. At the same age, the level of glycerophospholipids in the brains of AD patients is lower than that of the control group (Kim et al., 2014). These alterations in glycerophospholipids lead to changes in cell membrane permeability and ionic homeostasis, which also contribute to oxidative stress and neurodegenerative changes (Sagy-Bross et al., 2015).

It is worth noting that the enrichment analysis of the gut microbes and differential metabolites in the hippocampus suggests that ApoE may affect cognitive dysfunction in aging mice by regulating lipid metabolism. Lipids are one of the basic components of neuronal cell membranes and are closely related to the normal metabolism and abnormal accumulation of Aβ (Ehehalt et al., 2003). Many studies have shown that lipid metabolism plays an important role in the process of aging and gradual cognitive dysfunction (Johnson and Stolzing, 2019; Wei et al., 2020; Mutlu et al., 2021). In this study, we found that the serum concentrations of TC, TG, and LDL were significantly increased in aging mice, while ApoE deletion aggravated dyslipidemia in aging mice. Clinical studies have shown that higher serum concentrations of TC and LDL-C and a higher LDL-C/HDL-C ratio are positively correlated with cognitive decline (An et al., 2019). ApoE plays a crucial role in lipid metabolism (Aires et al., 2021). Another study of 1,065 individuals aged 56–105 found that centenarians had the highest ApoE plasma concentrations (Muenchhoff et al., 2017). The above studies suggest that ApoE may affect the cognitive function of aging mice by regulating lipid metabolism. Disorders of lipid metabolism are often accompanied by oxidative stress (Zarrouk et al., 2020), and oxidative stress is an important risk factor for aging and cognitive dysfunction (Mecocci et al., 2018). Abnormal oxidative stress can cause lipid peroxidation, leading to apoptosis and tissue damage, which is a major risk factor for various neurodegenerative diseases (Vatner et al., 2020). This study showed that the MDA content in the serum and hippocampus of aging mice was significantly increased, the activities of T-SOD and GSH-Px were significantly decreased, and ApoE knockout exacerbated oxidative stress in aging mice. Recently, the gut microbiota–brain axis has gained extensive attention as a channel for communication and physiological regulation. The activity of the gut microbiome may promote abnormal lipid deposition and oxidation reactions, which can damage the brain (Shao et al., 2020). This study speculated that ApoE knockout affected gut microbiota diversity in aging mice, aggravated disordered lipid metabolism and oxidative stress and exacerbated cognitive dysfunction in aging mice.

Notably, this study has some limitations. We did not use germ-free mice or fecal transplantation and could not determine exactly which flora are associated with the effects of ApoE. Second, there are several ApoE alleles (ApoE2, ApoE3, and ApoE4) in humans, and this study did not explore the effect of the corresponding alleles on aging. Additionally, the findings still need to be clinically validated and warrant further investigation. Moreover, we did not determine whether aging and ApoE deletion lead to altered lipid metabolism in the gut. We will continue this research in future work.

## Conclusion

In summary, this study demonstrated that ApoE deficiency aggravated cognitive function and hippocampal synaptic ultrastructural damage in aging mice, aggravated the dysregulation of hippocampal SYP and PSD-95 protein expression, affected the gut microbial composition and hippocampal metabolic profile in aging mice, and aggravated dyslipidemia and oxidative stress in aging mice. This study helps us to further reveal the underlying mechanism by which ApoE improves aging-related cognitive dysfunction through the gut microbiota–brain axis, which is worthy of further research.

## Data availability statement

The datasets presented in this study can be found in online repositories. The names of the repository/repositories and accession number (s) can be found in the article/Supplementary material.

## Ethics statement

This animal study was reviewed and approved by the Ethics Committee of the First Affiliated Hospital of Hunan University of Traditional Chinese Medicine.

## Author contributions

BC and JY: draft preparation, *in vitro* experiments, and writing the manuscript. YX: behavioral experiments and data analysis. HW, FT, and YL: model construct. LL and LX: data analysis. BL: experiment design and supervision. All authors contributed to the article and approved the submitted version.

## Footnotes

1
https://www.kegg.jp/

## Supplementary material

Supplementary Table 1 Differential metabolites identified by LC-MS.

Supplementary Table 2 Differential metabolites identified by GC-MS.

## References

[B2] Aires R. Porto M. de Assis L. M. Pereira P. Carvalho G. Côco L. (2021). DNA damage and aging on hematopoietic stem cells: Impact of oxidative stress in ApoE/mice. *Exp. Gerontol.* 156:111607. 10.1016/j.exger.2021.111607 34715304

[B3] An Y. Zhang X. Wang Y. Wang Y. Liu W. Wang T. (2019). Longitudinal and nonlinear relations of dietary and Serum cholesterol in midlife with cognitive decline: Results from EMCOA study. *Mol. Neurodegener.* 14:51. 10.1186/s13024-019-0353-1 31888696 PMC6937942

[B4] Azman K. Zakaria R. (2019). D-Galactose-induced accelerated aging model: An overview. *Biogerontology* 20 763–782. 10.1007/s10522-019-09837-y 31538262

[B5] Bell R. Winkler E. Singh I. Sagare A. Deane R. Wu Z. (2012). Apolipoprotein E controls cerebrovascular integrity via cyclophilin a. *Nature* 485 512–516. 10.1038/nature11087 22622580 PMC4047116

[B6] Blacher E. Bashiardes S. Shapiro H. Rothschild D. Mor U. Dori-Bachash M. (2019). Potential roles of gut microbiome and metabolites in modulating ALS in mice. *Nature* 572 474–480. 10.1038/s41586-019-1443-5 31330533

[B7] Cantuti-Castelvetri L. Fitzner D. Bosch-Queralt M. Weil M. Su M. Sen P. (2018). Defective cholesterol clearance limits remyelination in the aged central nervous system. *Science* 359 684–688. 10.1126/science.aan4183 29301957

[B8] Chakrabarti A. Geurts L. Hoyles L. Iozzo P. Kraneveld A. La Fata G. (2022). The microbiota-gut-brain axis: Pathways to better brain health. Perspectives on what we know, what we need to investigate and how to put knowledge into practice. *Cell. Mol. Life Sci.* 79:80. 10.1007/s00018-021-04060-w 35044528 PMC8770392

[B9] Chen B. Yi J. Xu Y. Zheng P. Tang R. Liu B. (2022). Construction of a circRNA-miRNA-mRNA network revealed the potential mechanism of Buyang Huanwu Decoction in the treatment of cerebral ischemia. *Biomed. Pharmacother.* 145:112445. 10.1016/j.biopha.2021.112445 34844103

[B10] Doifode T. Giridharan V. Generoso J. Bhatti G. Collodel A. Schulz P. (2021). The impact of the microbiota-gut-brain axis on Alzheimer’s disease pathophysiology. *Pharmacol. Res.* 164:105314. 10.1016/j.phrs.2020.105314 33246175

[B11] Ehehalt R. Keller P. Haass C. Thiele C. Simons K. (2003). Amyloidogenic processing of the Alzheimer beta-amyloid precursor protein depends on lipid rafts. *J. Cell Biol.* 160 113–123. 10.1083/jcb.200207113 12515826 PMC2172747

[B12] Emery D. C. Shoemark D. K. Batstone T. E. Waterfall C. M. Coghill J. A. Cerajewska T. L. (2017). 16S rRNA next generation sequencing analysis shows bacteria in alzheimer’s Post-Mortem brain. *Front. Aging Neurosci.* 9:195. 10.3389/fnagi.2017.00195 28676754 PMC5476743

[B13] Gan G. Lu B. Zhang R. Luo Y. Chen S. Lei H. (2022). Chronic apical periodontitis exacerbates atherosclerosis in apolipoprotein E-deficient mice and leads to changes in the diversity of gut microbiota. *Int. Endod. J.* 55 152–163. 10.1111/iej.13655 34714545 PMC9298730

[B14] Güldner F. Ingham C. (1980). Increase in postsynaptic density material in optic target neurons of the rat suprachiasmatic nucleus after bilateral enucleation. *Neurosci. Lett.* 17 27–31. 10.1016/0304-3940(80)90056-7 6302580

[B15] Huang J. Hou B. Zhang S. Wang M. Lu X. Wang Q. (2020). The protective effect of Adiponectin-Transfected endothelial progenitor cells on cognitive function in D-Galactose-Induced aging rats. *Neural Plast.* 2020:1273198. 10.1155/2020/1273198 32273888 PMC7125484

[B16] Hudry E. Klickstein J. Cannavo C. Jackson R. Muzikansky A. Gandhi S. (2019). Opposing Roles of apolipoprotein E in aging and neurodegeneration. *Life Sci. Alliance* 2:e201900325. 10.26508/lsa.201900325 30760557 PMC6374993

[B17] Hui S. Yang Y. Peng W. Sheng C. Gong W. Chen S. (2017). Protective effects of *Bushen Tiansui* decoction on hippocampal synapses in a rat model of Alzheimer’s disease. *Neural Regen. Res.* 12 1680–1686. 10.4103/1673-5374.217347 29171433 PMC5696849

[B18] Joe E. Ringman J. (2019). Cognitive symptoms of Alzheimer’s disease: Clinical management and prevention. *BMJ* 367:l6217. 10.1136/bmj.l6217 31810978

[B19] Johnson A. Stolzing A. (2019). The role of lipid metabolism in aging, lifespan regulation, and age-related disease. *Aging Cell* 18:e13048. 10.1111/acel.13048 31560163 PMC6826135

[B20] Jones D. Devon R. (1978). An ultrastructural study into the effects of pentobarbitone on synaptic organization. *Brain Res.* 147 47–63. 10.1016/0006-8993(78)90771-0 207385

[B21] Khine W. Voong M. Ng T. Feng L. Rane G. Kumar A. (2020). Mental awareness improved mild cognitive impairment and modulated gut microbiome. *Aging* 12 24371–24393. 10.18632/aging.202277 33318317 PMC7762482

[B22] Kim K. Jeong J. Yoo S. Kim D. (2016). Gut microbiota lipopolysaccharide accelerates inflamm-aging in mice. *BMC Microbiol.* 16:9. 10.1186/s12866-016-0625-7 26772806 PMC4715324

[B23] Kim S. Cheon H. Song J. Yun S. Park S. Jeon J. (2014). Aging-related changes in mouse serum glycerophospholipid profiles. *Osong Public Health Res. Perspect.* 5 345–350. 10.1016/j.phrp.2014.10.002 25562043 PMC4281626

[B24] Kodali M. Attaluri S. Madhu L. Shuai B. Upadhya R. Gonzalez J. (2021). Metformin treatment in late middle age improves cognitive function with alleviation of microglial activation and enhancement of autophagy in the hippocampus. *Aging Cell* 20:e13277. 10.1111/acel.13277 33443781 PMC7884047

[B25] Kokudai Y. Honma M. Masaoka Y. Yoshida M. Sugiyama H. Yoshikawa A. (2021). Cascade process mediated by left hippocampus and left superior frontal gyrus affects relationship between aging and cognitive dysfunction. *BMC Neurosci.* 22:75. 10.1186/s12868-021-00680-x 34876001 PMC8650545

[B26] Li B. He Y. Ma J. Huang P. Du J. Cao L. (2019). Mild cognitive impairment has similar alterations as Alzheimer’s disease in gut microbiota. *Alzheimers Dement.* 15 1357–1366. 10.1016/j.jalz.2019.07.002 31434623

[B27] Liu X. Tang S. Zhong H. Tong X. Jie Z. Ding Q. (2021b). A genome-wide association study for gut metagenome in Chinese adults illuminates complex diseases. *Cell Discov.* 7:9. 10.1038/s41421-020-00239-w 33563976 PMC7873036

[B28] Liu X. Zhao Y. Zhu H. Wu M. Zheng Y. Yang M. (2021c). Taxifolin retards the D-galactose-induced aging process through inhibiting Nrf2-mediated oxidative stress and regulating the gut microbiota in mice. *Food Funct.* 12 12142–12158. 34788354 10.1039/d1fo01349a

[B29] Liu C. Cheng Y. Guo Y. Qian H. (2021a). Magnesium-L-threonate alleviate colonic inflammation and memory impairment in chronic-plus-binge alcohol feeding mice. *Brain Res. Bull.* 174 184–193. 10.1016/j.brainresbull.2021.06.009 34144203

[B30] Luo D. Chen K. Li J. Fang Z. Pang H. Yin Y. (2020). Gut microbiota combined with metabolomics reveals the metabolic profile of the normal aging process and the anti-aging effect of FuFang Zhenshu TiaoZhi(FTZ) in mice. *Biomed. Pharmacother.* 121:109550. 10.1016/j.biopha.2019.109550 31704617

[B31] Mangiola F. Nicoletti A. Gasbarrini A. Ponziani F. (2018). Gut microbiota and aging. *Eur. Rev. Med. Pharmacol.* 22 7404–7413. 10.26355/eurrev_201811_16280 30468488

[B32] Mariat D. Firmesse O. Levenez F. Guimarães V. Sokol H. Doré J. (2009). The Firmicutes/Bacteroidetes ratio of the human microbiota changes with age. *BMC Microbiol.* 9:123. 10.1186/1471-2180-9-123 19508720 PMC2702274

[B33] Mecocci P. Boccardi V. Cecchetti R. Bastiani P. Scamosci M. Ruggiero C. (2018). A long journey into aging, brain aging, and alzheimer’s disease following the oxidative stress tracks. *J. Alzheimers Dis.* 62 1319–1335. 10.3233/JAD-170732 29562533 PMC5870006

[B34] Mou Y. Du Y. Zhou L. Yue J. Hu X. Liu Y. (2022). Gut microbiota interact with the brain through systemic chronic inflammation: Implications on neuroinflammation, neurodegeneration, and aging. *Front. Immunol.* 13:796288. 10.3389/fimmu.2022.796288 35464431 PMC9021448

[B35] Muenchhoff J. Song F. Poljak A. Crawford J. Mather K. Kochan N. (2017). Plasma apolipoproteins and physical and cognitive health in very old individuals. *Neurobiol. Aging* 55 49–60. 10.1016/j.neurobiolaging.2017.02.017 28419892

[B36] Mulder M. Blokland A. van den Berg D. J. Schulten H. Bakker A. Terwel D. (2001). Apolipoprotein E protects against neuropathology induced by a high-fat diet and maintains the integrity of the blood-brain barrier during aging. *Lab. Invest.* 81 953–960. 10.1038/labinvest.3780307 11454984

[B37] Mutlu A. Duffy J. Wang M. (2021). Lipid metabolism and lipid signals in aging and longevity. *Dev. Cell* 56 1394–1407. 10.1016/j.devcel.2021.03.034 33891896 PMC8173711

[B38] Pw O. Jeffery I. (2015). Gut microbiota and aging. *Science* 350 1214–1215. 10.1126/science.aac8469 26785481

[B39] Rajeswari T. S. Radha E. (1984). Metabolism of the glutamate group of amino acids in rat brain as a function of age. *Mech. Ageing Dev.* 24 139–149. 10.1016/0047-6374(84)90066-6 6143862

[B40] Rowland I. Gibson G. Heinken A. Scott K. Swann J. Thiele I. (2018). Gut microbiota functions: Metabolism of nutrients and other food components. *Eur. J. Nutr.* 57 1–24. 10.1007/s00394-017-1445-8 28393285 PMC5847071

[B41] Sagy-Bross C. Kasianov K. Solomonov Y. Braiman A. Friedman A. Hadad N. (2015). The role of cytosolic phospholipase A2 α in amyloid precursor protein induction by amyloid beta1-42 : Implication for neurodegeneration. *J. Neurochem.* 132 559–571. 10.1111/jnc.13012 25533654

[B42] Saji N. Murotani K. Hisada T. Tsuduki T. Sugimoto T. Kimura A. (2019). The relationship between the gut microbiome and mild cognitive impairment in patients without dementia: A cross-sectional study conducted in Japan. *Sci. Rep.* 9:19227. 10.1038/s41598-019-55851-y 31852995 PMC6920432

[B43] Shao A. Lin S. Wang L. Tu S. Lenahan C. Zhang J. (2020). Oxidative stress at the crossroads of aging, stroke and depression. *Aging Dis.* 11 1537–1566. 10.14336/AD.2020.0225 33269106 PMC7673857

[B44] Sheng S. Yang J. Xu Y. Kong X. Wang J. Wang Y. (2022). Alleviation effects of grape seed proanthocyanidin extract on inflammation and oxidative stress in a D-galactose-induced aging mouse model by modulating the gut microbiota. *Food Funct.* 13 1348–1359. 10.1039/d1fo03396d 35043135

[B45] Sibai M. Altuntaş E. Yıldırım B. Öztürk G. Yıldırım S. Demircan T. (2020). Spalax leucodonMicrobiome and longevity: High abundance of Longevity-Linked muribaculaceae in the gut of the Long-Living rodent. *OMICS* 24 592–601. 10.1089/omi.2020.0116 32907488

[B46] Suryavanshi P. Ugale R. Yilmazer-Hanke D. Stairs D. Dravid S. (2014). GluN2C/GluN2D subunit-selective NMDA receptor potentiator CIQ reverses MK-801-induced impairment in prepulse inhibition and working memory in Y-maze test in mice. *Br. J. Pharmacol.* 171 799–809. 10.1111/bph.12518 24236947 PMC3969090

[B47] Vatner S. Zhang J. Oydanich M. Berkman T. Naftalovich R. Vatner D. (2020). Healthful aging mediated by inhibition of oxidative stress. *Ageing Res. Rev.* 64:101194. 10.1016/j.arr.2020.101194 33091597 PMC7710569

[B48] Wang Y. Kuang Z. Yu X. Ruhn K. Kubo M. Hooper L. (2017). The intestinal microbiota regulates body composition through NFIL and the circadian clock. *Science* 357 912–916. 10.1126/science.aan0677 28860383 PMC5702268

[B49] Watson Y. Nelson B. Kluesner J. Tanzy C. Ramesh S. Patel Z. (2021). Aggregate trends of apolipoprotein e on cognition in transgenic alzheimer’s disease mice. *J. Alzheimers Dis.* 83 435–450. 10.3233/JAD-210492 34334405 PMC8461675

[B50] Wei S. Gao L. Jiang Y. Shang S. Chen C. Dang L. (2020). The apolipoprotein e ε4 Allele-Dependent relationship between serum lipid levels and cognitive function: A Population-Based cross-sectional study. *Front. Aging Neurosci.* 12:44. 10.3389/fnagi.2020.00044 32231559 PMC7082227

[B51] Wu L. Liu X. Hu R. Chen Y. Xiao M. Liu B. (2022). Agrocybe cylindraceaPrebiotic crude polysaccharides combined with GG postpone aging-related oxidative stress in mice. *Food Funct.* 13 1218–1231. 10.1039/d1fo02079j 35019929

[B52] Yang X. Yu D. Li X. Li H. Du J. (2020). Probiotics modulate the microbiota–gut–brain axis and improve memory deficits in aged SAMP8 mice. *Acta Pharm. Sin. B* 10 475–487. 32140393 10.1016/j.apsb.2019.07.001 PMC7049608

[B53] Zajac D. Green S. Johnson L. Estus S. (2022). APOE genetics influence murine gut microbiome. *Sci. Rep.* 12:1906. 10.1038/s41598-022-05763-1 35115575 PMC8814305

[B54] Zarrouk A. Hammouda S. Ghzaiel I. Hammami S. Khamlaoui W. Ahmed S. (2020). Association between oxidative stress and altered cholesterol metabolism in alzheimer’s disease patients. *Curr. Alzheimer Res.* 17 823–834. 10.2174/1567205017666201203123046 33272182

[B55] Zerbi V. Wiesmann M. Emmerzaal T. Jansen D. Van Beek M. Mutsaers M. (2014). Resting-state functional connectivity changes in aging apoE4 and apoE-KO mice. *J. Neurosci.* 34 13963–13975. 10.1523/JNEUROSCI.0684-14.2014 25319693 PMC6705289

[B56] Zhao T. Zhong S. Xu J. Jiao W. Liu W. Huang L. (2022). PAYCS alleviates Scopolamine-Induced memory deficits in mice by reducing oxidative and inflammatory stress and modulation of gut Microbiota-Fecal Metabolites-Brain neurotransmitter axis. *J. Agr. Food Chem.* 70 2864–2875. 10.1021/acs.jafc.1c06726 35174709

